# Impact of acute hyperglycemic crisis episode on survival in individuals with diabetic foot ulcer using a machine learning approach

**DOI:** 10.3389/fendo.2022.974063

**Published:** 2022-08-25

**Authors:** Liling Deng, Puguang Xie, Yan Chen, Shunli Rui, Cheng Yang, Bo Deng, Min Wang, David G. Armstrong, Yu Ma, Wuquan Deng

**Affiliations:** ^1^ Department of Endocrinology and School of Medicine, Chongqing University Central Hospital, Chongqing University, Chongqing, China; ^2^ Department of Surgery, Keck School of Medicine of University of Southern California, Los Angeles, CA, United States

**Keywords:** hyperglycemic crisis episode, diabetic foot ulcer, machine learning, mortality rates, risk factors of mortality

## Abstract

**Objective:**

The outcome of DFUs concomitant with HCE remains unknown. This study aimed to investigate mortality rates and identify risk factors of mortality in patients with DFUs-HCE.

**Methods:**

27 inpatients with DFUs-HCE were retrospectively enrolled in a cohort design, they were compared to 93 inpatients with DFUs in a city designated emergency center, between January 2016 and January 2021. After a 6-year followed-up, clinical characteristic, amputation and survival rates were compared. Extreme gradient boosting was further used to explore the relative importance of HCE and other risk factors to all-cause mortality in DFUs.

**Results:**

Patients with DFUs-HCE were more likely to havedementia, acute kidney injury and septic shock, whereas DFUs were more likely to have diabetic peripheral neuropathy and ulcer recurrence (P<0.05). No significant difference was observed on the amputation rate and diabetes duration. Both Kaplan-Meier curves and adjusted Cox proportional model revealed that DFUs-HCE was associated with a higher mortality compared with DFUs (P<0.05). HCE significantly increased the risk of mortality in patients with DFUs (hazard ratio, 1.941; 95% CI 1.018-3.700; P = 0.044) and was independent from other confounding factors (age, sex, diabetes duration, Wagner grades and Charlson Comorbidity Index). The XGBoost model also revealed that HCE was one of the most important risk factors associated with all-cause mortality in patients with DFUs.

**Conclusions:**

DFUs-HCE had significantly lower immediate survival rates (first 1-6 month) than DFUs alone. HCE is an important risk factor for death in DFUs patients.

## Introduction

Diabetic foot ulcers (DFUs), a common and severe complication of diabetes characterized by lesions in the deep tissues associated with neurological disorders and peripheral arterial disease (PAD) in the lower limbs, which exerts a substantial impact on disability, morbidity and mortality (1). Previous studies have estimated that up to 6% diabetic patients worldwide will develop DFUs over the course of their lifetime (2). On the other hand, about 85% of nontraumatic lower-extremity amputations are preceded by diabetic foot ulceration. Although DFUs usually manifest as a topical clinical phenomenon, clinical outcomes of patients is mainly depended on systematic comorbidities or complications. Previous studies have shown that 5-year mortality for DFUs are comparable to those reported in cancer (3). In addition, patients with chronic kidney disease and dialysis had more minor and major amputations, albeit at significantly lower rates than survival (4).

DFUs commonly manifest as chronic or acute ischemic diabetic foot. However, the particular raising presentation stands out for its severity and need for emergency treatment, which termed “diabetic foot attack” (DFA). It is the most critical devastating presentations of diabetic foot ulcers typically presenting as an acutely inflamed foot with rapidly progressive soft and bone tissue necrosis, associated with severe systemic symptoms, such as hyperthermia, septic shock and so on (5). Without timely intervention, it may deteriorate over hours and poses a high amputation and life-threatening risk. It is well known that severe infections, including acute diabetic foot wounds infection, can induce acute hyperglycemic crisis episode (HCE), such as diabetic ketoacidosis (DKA) and hyperglycemic hyperosmolar status (HHS) (6).

HCE, a disease continuum of most severe acute diabetes-related complication, including DKA, HHS and mixed syndrome (a mixed state of acidosis and hyperosmolality), is mostly caused by severe blood glucose fluctuations, infections or other predisposing factors (6). It is also associated with significant in-hospital morbidity and mortality (7, 8). HCE patients were found to have a mortality rate of up to 10% in China (9), while those with HHS exhibited higher mortality rates (10). Moreover, HCE is associated with increased risk of end-stage renal disease (11).

However, up to date, studies about DFUs concomitant with HCE are still scarce, especially the impact of HCE on the prognosis of DFUs patients, and almost all focus on isolated DFUs or HCE. In addition, previous studies exploring risk factors associated with disease mainly relied on analysis *via* Logistic regression model or Cox proportional hazard model. Although these models are commonly used statistical analysis methods and can provide information about risk factors, they failed to capture the complex non-linear relationships between the risk factors, leading to the decline of the prediction ability of the model and the inability to accurately assess the relative importance of risk factors. Machine learning opens up a new horizon for analyzing the risk factors of disease, which can solve the limitations of traditional linear models, verify the analysis results of linear models, and improve the accuracy of analysis (12–14).

In this study, we aimed to investigate clinical features and amputation rates, analyze the mortality and identify possible risk factors of all-cause mortality of HCE overlapping in DFUs patients. Additionally, we further explore the relative importance of HCE and other risk factors to all-cause mortality in patients with DFUs based on machine learning methods.

## Subjects and methods

### Study subjects

A total of 27 inpatients with DFUs concomitant with HCE were retrospectively enrolled in a cohort design, and compared to 93 inpatients with isolated DFUs in a city designated emergency center, between January 2016 and January 2021. Patients with diabetes had a higher mortality risk after HCE during the first 6 years of follow-up (8, 15). All patients were therefore followed up to the date of death or 1 July 2021. Baseline characteristics, laboratory parameters and Charlson Comorbidity Index (CCI) (16) were recorded during the follow-up. All study patients were type 2 diabetes. The main outcome of interest was hospitalization or long-term mortality.

Diabetic wounds were graded using Wagner’s classification. We defined a foot infection by clinical criteria according to the International Working Group guidelines. We evaluated patients with an infection for the extent of soft tissue involvement and for evidence of bone involvement. Bone infection was confirmed in the presence of positive probe-to bone test (17)or *via* magnetic resonance imaging (MRI). The criteria for the diagnosis of HCE were based on the 2009 American Diabetes Association criteria (18). DKA comprises a triad of hyperglycemia, hyperketonemia and metabolic acidosis (plasma glucose 13.9 mmol/L, positive urine or serum ketone, arterial pH<7.3, and serum bicarbonate<18mEq/L).The diagnostic criteria for HHS include a plasma glucose level>33.3mmol/L, and effective serum osmolality >320mmol/kg and the absence of metabolic acidosis and ketonemia. This study was approved by the Ethics Committee of Chongqing University Central Hospital (Clinical Trial Registration Number: ChiCTR1800019179). All subjects provided informed consent prior to enrollment.

### Statistical analysis

Descriptive analyses were conducted for both DFUs alone group and DFUs-HCE group. The continuous data were presented as mean ± standard deviation and median (interquartile range) according to whether the variables obeyed normal distribution or not. Normality of variables were tested using the Shapiro-Wilk test. The categorical data are expressed as percentages. The Mann-Whitney U test was used for the comparisons of the continuous variables. The chi-squared test or Fisher’s exact test was used to analyze the differences in the categorical variables between two independent groups as appropriate. The log-rank test was used to test the difference of Kaplan-Meier survival between the DFUs alone group and DFUs-HCE group. Cox proportional hazards model adjusted for age, sex, diabetes duration, Wagner grades and CCI was used to assess the impact of HCE on mortality in patients with DFUs. A P value<0.05 was considered statistically significant. The statistical analysis was conducted using SPSS version 26.0.

### Model development and evaluation

Extreme gradient boosting (XGBoost) algorithm is an efficient and flexible machine learning method with excellent scalability and a high running speed based on gradient tree boosting, which can learn nonlinear, high dimensional relationships from data (19, 20). XGBoost often outperforms other machine learning algorithms for prediction with tabular data, and is currently considered as the state-of-the-art method for predicting tabular data (21, 22). Therefore, XGBoost was used to predict mortality and assess the relative importance of potential risk factors in the study population. The whole dataset was randomly split into a training set (70%) and a test set (30%). We performed 3-fold cross-validation and grid-search on the training set to obtain optimized parameter sets and train the model. In 3-fold cross-validation, the training set was randomly split into 3 equal-sized subsets, and each subset was selected as the testing set in turn while other subsets were selected as training subsets and used to train the model. We then evaluated the performance of the developed XGBoost model on the testing set using four commonly used evaluation metrics; area under the receiver-operating-characteristic curve (AUC), accuracy, sensitivity, specificity. In addition, we used the developed XGBoost model to calculate the important scores of features and assess the relative importance of the risk factors according to the scores. Model development and evaluation were performed using standard Python packages (Python 3.6.1).

## Results

### Baseline characteristics of participants of DFUs with and without HCE

Among 120 inpatients in the two groups, males exhibited a significantly higher DFU incidence. No significant difference was observed on the mean age, amputation rate and diabetes duration. Among the 27 cases of DFUs concomitant with HCE, 23 cases of DFUs preceded the onset of HCE, whereas 4 of them developed HCE firstly. Compared with isolated DFUs patients, DFUs-HCE were more likely to have a higher incidence of dementia, acute kidney injury and septic shock. Moreover, 6 (22.2%) patients in the DFUs concomitant with HCE group were first diagnosed with septic shock on admission, while only 3 (3.2%) in the isolated DFUs group developed septic shock. In contrast, patients with isolated DFUs exhibited a higher incidence of diabetic peripheral neuropathy. In addition, there was no significant difference in the endovascular intervention rate and age of wounds in the two groups (**Table 1**).

**Table 1:** Baseline characteristics of patients with DFUs by HCE.

Variables	Combined HCE (n = 27)	DF (n = 93)	*P* value
Demographic data			
Age, years	68.7 ± 14.3	69.3 ± 14.7	0.967
Sex			0.326
Male, %	85.2	76.3	
Female, %	14.8	23.7	
Diabetes duration, years	8.00 (1.00-20.00)	10.0 (4.0-17.0)	0.140
Medical comorbidity			
Deep vein thrombosis, %	22.2	10.8	0.123
COPD, %	0.0	8.6	0.115
Septic shock, %	22.2	3.2	0.001
PAD, %	66.7	62.4	0.683
DPN, %	48.1	81.7	<0.001
Infection other than foot wounds, %	59.3	40.9	0.099
Dementia, %	18.5	4.3	0.014
Cardiovascular disease, %	55.6	71.0	0.132
Stroke, %	25.9	31.2	0.600
Cancer, %	0.0	5.4	0.218
CKD			0.466
Stage I, %	0.0	0.0	
stage II, %	0.0	2.2	
stage III, %	3.7	11.8	
stage IV, %	7.4	5.4	
stage V, %	3.7	9.7	
AKI, %	44.4	7.5	<0.001
Vascular intervention, %	7.4	10.8	0.686
Age of the wounds, days	12.0 (7.0, 30.0)	29.0 (10.0-60.0)	0.073
Clinical and laboratory data			
HbA1c, %	12.0 ± 2.9	8.90 ± 2.26	<0.001
HbA1c, mmol/mol	108.0 ± 26.1	74.0 ± 18.8	<0.001
β-hydroxybutyrate, μmol/L	3.49 (0.80-6.50)	0.30 (0.20-0.60)	<0.001
HCO3−, mmol/L	16.3 (10.8-19.5)	0.00 (0.00-23.75)	0.008
Hemoglobin, g/L	118.5 (89.5-138.5)	111.0 (88.0-127.0)	0.149
Platelet, 109/L	246.5 ± 117.1	257.4 ± 109.1	0.634
CRP, mg/L	136.1 (63.0-230.5)	32.4 (9.8-127.1)	<0.001
Serum albumin, g/L	27.8 ± 5.6	34.6 ± 7.3	<0.001
Serum potassium, mmol/L	4.10 (3.48-5.10)	4.08 (3.70-4.38)	0.598
Serum sodium, mmol/L	136.3 ± 9.4	137.2 ± 4.4	0.298
Lactic acid, mmol/L	2.74 (2.19-4.44)	2.29 (1.82-2.87)	0.021
Osmolality, mmol/L	330.0 (300.0-342.0)	298.8 (288.8-306.3)	<0.001
Classification systems			
Wagner Classification System			0.222
0, %	3.7	1.1	
1, %	0.0	10.8	
2, %	7.4	16.1	
3, %	29.6	22.6	
4, %	29.6	32.3	
5, %	29.6	17.2	
Charlson Comorbidity Index	4.00 (3.00-5.00)	4.00 (3.00-5.00)	0.168

DFUs, diabetic foot ulcers; HCE, hyperglycemic crisis episode; COPD, chronic obstructive.

pulmonary disease; PAD, peripheral artery disease; DPN, diabetic peripheral neuropathy; CKD, chronic kidney disease; AKI, acute kidney injury; HbA1c, hemoglobin A1c; CRP, C-reactive. protein. P < 0.05 was considered statistically significant (Bold values indicate P < 0.05).

### DFU recurrence

**Figure 1:**
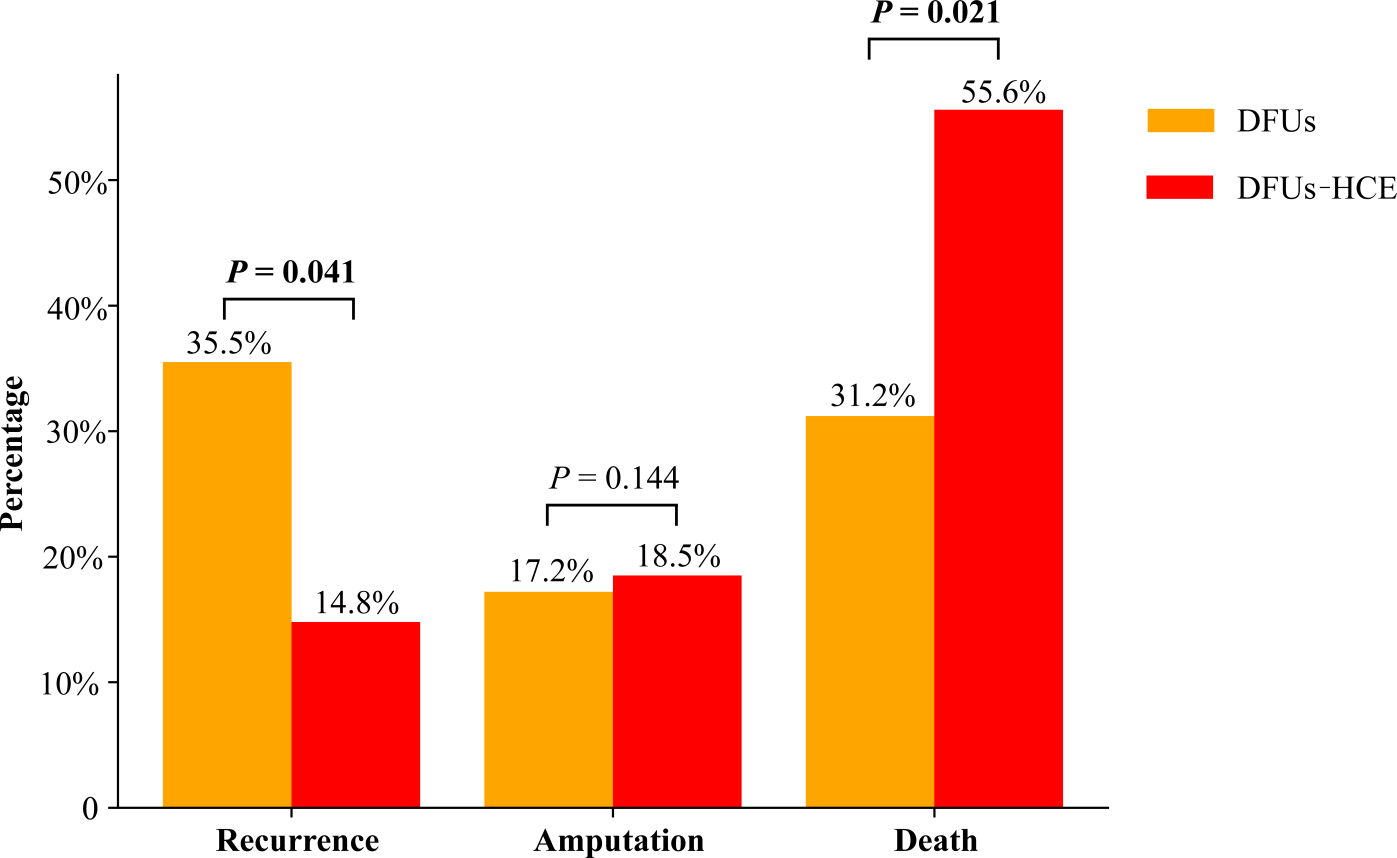
Clinical outcomes of patients with DFU’s by HCE.

Patients with isolated DFUs exhibited a higher incidence of ulcer recurrence during the follow-up period compared to the DFUs-HCE group. Notably, 33 (35.5%) patients in the DFUs alone group developed a new ulcer, however, 4 (14.8%) developed a new ulcer in DFUs-HCE group (**Figure 1**).

### Amputation

There was no significant difference in the amputation rate of DFUs between the two groups(**Figure 1**).

### Mortality

**Figure 2:**
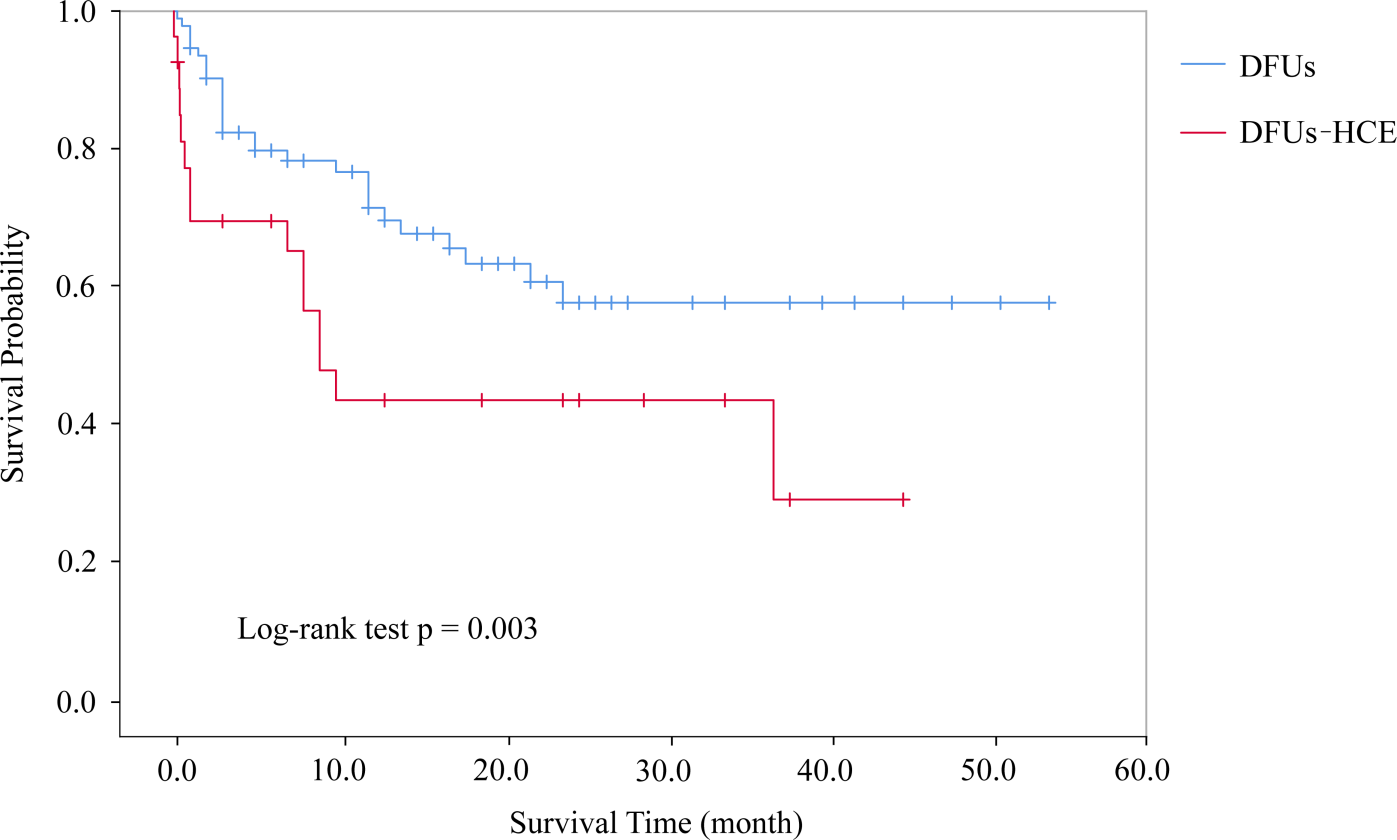
Cumulative Kaplan-Meier survival curves.

A total of 15 patients in the DFUs-HCE group died over the course of the follow-up period, including 5 from septic shock caused by DFUs, 3 from the cardiovascular events, 3 from chronic kidney disease, and 4 from other causes. On the other hand, 29 patients died in the DFUs group during the follow-up, including 13 from the cardiovascular events, 4 from chronic kidney disease, 2 from malignancies, and 10 from other causes (**Table 2**). Patients with DFUs-HCE had a significantly higher mortality compared with patients with isolated DFUs (55.6% vs. 31.2%, P<0.05). Kaplan-Meier survival analysis and log-rank tests showed that DFUs-HCE group patients had significantly lower survival rates than DFUs alone subjects, especially declined rapidly during the first month of follow-up **(** **Figure 2**). During the first month after discharge, the survival rate of DFUs-HCE group patients was 69.4% whereas 94.6% in the isolated DFUs group in the first month. Six months after discharge, the survival rate of DFUs-HCE group declined to 65.1%, whereas 78.2% in DF alone group. At 12 months after discharge, the survival rate of patients in the DFUs-HCE group declined to 43.4%, whereas 71.4% in DFUs alone group. At 36 months after discharge, the survival rate of patients in the DFUs-HCE and DF alone groups had declined to 28.9and 57.6%, respectively. Moreover, Adjusted Cox proportional hazards model showed that HCE increased the risk of mortality in patients with DFUs (hazard ratio, 1.941; 95% CI 1.018-3.700; *P* = 0.044), and was independent from other confounding factors (age, sex, diabetes duration, Wagner grades and CCI).

**Table 2:** Cause of death among patients with DFUs by HCE.

Cause of death	DFUs-HCE (n = 15)	DFUs (n = 29)	Total (n = 44)
Cardiovascular events, n (%) CKD class 5	3 (18.8) 3(42.9)	13 (81.2) 4(57.1)	16 7
Septic shock caused by DFUs, n (%)	5 (100.0)	0 (0.0)	5
Tumor, n (%)	0 (0.0)	2 (100.0)	2
Other, n (%)	4 (28.6)	10 (71.4)	14

DFUs, diabetic foot ulcers; CKD class 5, chronic kidney disease class 5;HCE, hyperglycemic crisis episode.

### Relative importance of risk factors for all-cause mortality in patients by XGBoost

**Figure 3:**
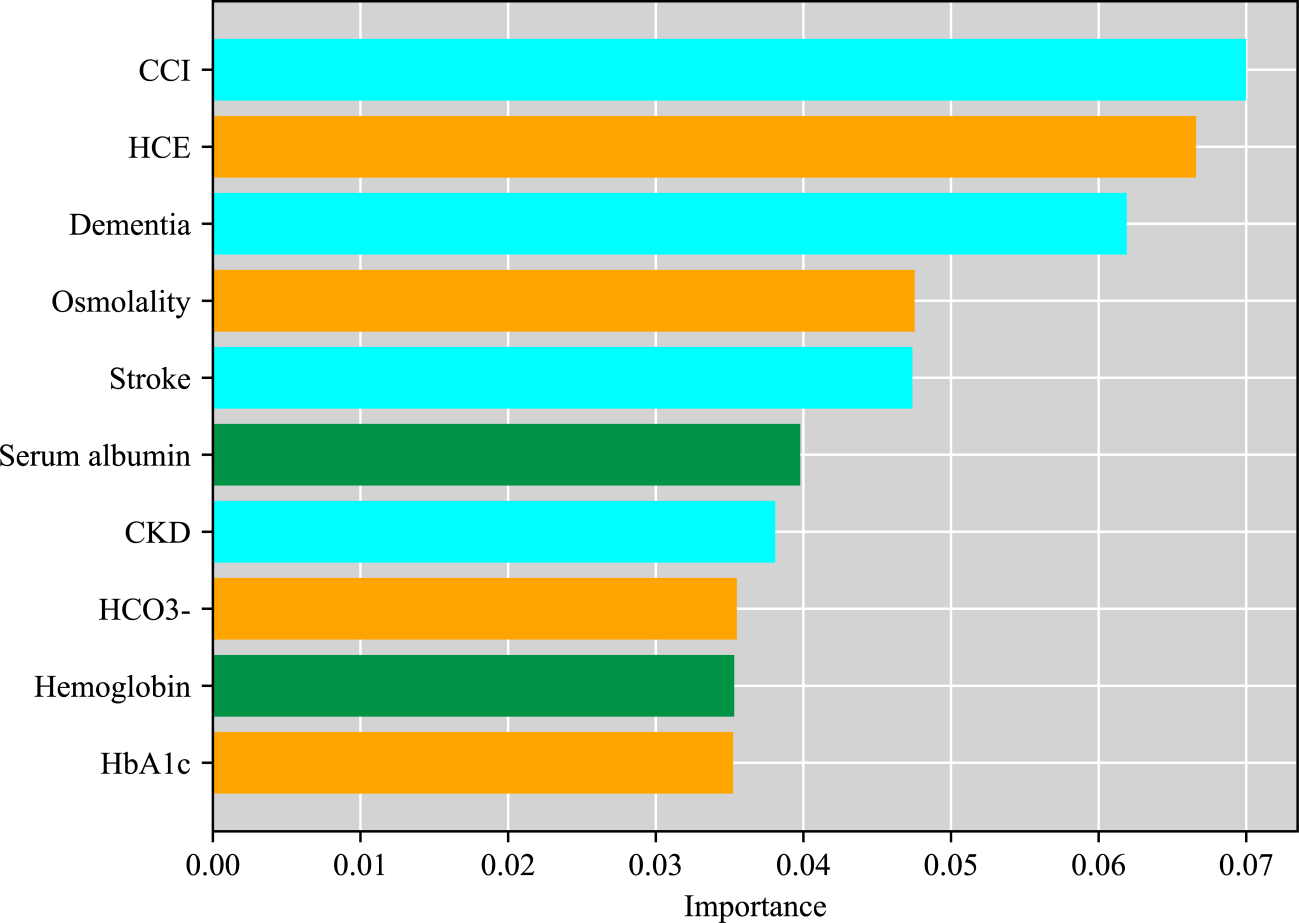
Relative importance of risk factors of all-cause mortality in patients with DFUs by XGBoost.

We developed an advanced machine learning model (XGBoost) to investigate risk factors of all-cause mortality of DFUs patients and evaluate relative importance of potential risk factors. The AUC, accuracy, sensitivity, and specificity of the developed model were 0.68, 0.69, 0.54, and 0.78, respectively, which proves that the developed model was efficient and can be used for risk factors analysis. The top ten features were selected according to the importance scores obtained from the developed XGBoost model. The higher the score, the more important the risk factors (**Figure 3**). Among all the risk factors for mortality of DFUs patients in this study, CCI was ranked first, followed by combined HCE, dementia, osmolality, stroke, serum albumin, chronic kidney disease, HCO3-, hemoglobin and HbA1c.

## Discussion

This is the first study to report DFUs combined with HCE and use a variety of statistical methods to evaluate the impact of HCE on the all-cause mortality of DFUs patients. Patients with DFUs-HCE had a higher incidence of acute kidney injury and septic shock compared to those in DFUs alone group, possibly due to severe dehydration, hyperviscosity, hypotension, or cholesterol embolism of HCE. In addition, patients in the DFUs-HCE group had a higher proportion of dementia in DFUs-HCE was also found, which was consistent with previous studies. HCE increased the risk of dementia in diabetic patients aged 45-64 years *via* microvascular and macrovascular injuries (23). However, isolated DFUs exhibited a higher incidence of diabetic peripheral neuropathy and ulcer recurrence. There was no significant difference in the amputation rate of DFUs between the two groups, and the amputation rates in our study was similar to that reported in a multicenter large cohort prospective study (EDI-FOCUS investigators) (24). Kaplan-Meier survival analysis and log-rank tests showed that patients in the DFUs-HCE group had significantly lower survival rates than those with DFUs alone, especially declined rapidly in the first month of follow-up period. In addition, the cumulative survival rates at years 1 and 3 in this research was remarkably lower than that reported in the other two long-term studies (24, 25), it may because our units were a critical care center, DFUs patients with/without HCE included were critically ill. Previous studies have shown that acute lower limb ischemia due to vascular embolism is a frequent complication of severe HHS or DKA, which may be related to dehydration and blood hyperviscosity (26, 27), that further aggravates DFU and causes vascular embolism diseases such as cerebral or myocardial ischemia infarction. In the present study, among the 27 cases of DFUs-HCE included in this study, 23 DFU cases induced to HCE, 12 of 23 (52%) cases were DFA, and 5 cases died from diabetic wound related septic shock, which further verified that DFA was the trigger of HCE. This may explain the higher mortality rates observed in patients with DFUs combined with HCE.

Diabetic foot ulcers mostly present as chronic or acute ischemic wounds with or without infection initially. However, without timely intervention of severe ischemia, progressive tissue damage and necrosis or infection may occur. DFA is the most critical manifestation of diabetic foot ulcers. Typically, the condition presents as an acutely inflamed foot with progressive soft and bone tissue necrosis, associated with severe systemic complications, such as hyperglycemic crisis, acute cardiac or cerebrovascular event, and septic shock and so on. Therefore, immediate awareness and urgent aggressive management is of more than vital importance to improve limb and patient survival.

DFUs and HCE are the severe complications of diabetes with higher rates of morbidity and mortality. Although isolated DFUs or HCE are highly prevalent, DFUs concomitant with HCE is relatively rare, which results in a lower incidence but with important clinical significance. Therefore, based on the above-mentioned epidemiological and clinical features, in order to improve the accuracy of analysis, in addition to conventional KM curve and Cox regression analyses, we developed an advanced machine learning model based on XGBoost to further assess the relative importance of risk factors for all-cause mortality in DFUs patients.

XGBoost is a machine learning method belonging to the optimized implementation of gradient boosting, which is highly efficient and flexible (19). In addition, XGBoost is able to capture the potential nonlinear and high-order relationships among the risk factors which can not be captured by common linear models such as Logistic regression model and Cox proportional hazard model. Moreover, XGBoost can automatically deal with the missing values and effectively assess the relative importance of risk factors without normalizing the data like common linear models. Since XGBoost overcomes the challenges presented by traditional liner models and evaluates the relative importance of risk factors more accurately and comprehensively, we used XGBoost to assess relative importance of risk factors for all-cause mortality in DFUs patients in addition to using Kaplan-Meier curves and adjusted Cox proportional hazards model to evaluate the impact of HCE on mortality in patients with DFUs. All the analytical results showed consistency, which increased the reliability of our analysis and indicated that HCE was one of the most important risk factors associated with all-cause mortality in patients with DFUs.

In this study, we selected the top ten features based on their contribution to the model and discussed them focusing on three parts, including patient general comorbidities, the humoral environment and nutritional status. Firstly, we found that CCI, dementia, stroke and CKD were the indicators that reflected the patients’ general medical conditions in the model. CCI was ranked first, which was a classic method of predicting 10-year survival in patients with multiple systemic comorbidities. Elderly dementia patients, who lost self-care ability, were at a higher risk of developing hyperglycemia and diabetes complications, such as HCE and DFUs. Additionally, amputation, microvascular diseases and glycemic control were also associated with impaired cognitive function in elderly diabetic patients (28), and the detrimental effects of hyperglycemia may not vanish immediately after correction of hyperglycemia (29). On the other hand, diabetic foot patients usually combined with cerebral artery stenosis, causing subsequent ischemic stroke. Furthermore, it was demonstrated that HCE was associated with an increased risk of subsequent ischemic stroke (30), which might be induced by severe dehydration, the hypercoagulable state, vascular thrombosis and inflammation. It is reasonable to infer that stroke might contribute to the death of DFUs patients with or without HCE.

Ndip et al. (4, 31) demonstrated that patients with CKD and dialysis had more below-knee amputations, above knee amputations and lower survival than those with no renal disease. Additional research evidence has shown that CKD is a formidable risk factor for poor intermediate outcomes after infrainguinal revascularization in diabetic patients with foot ulcer or gangrene (32). Moreover, CKD patients probably have severe diabetes-related comorbidities, so a high-level amputation and mortality may reflect the severity of peripheral vascular disease, neuropathy, cardiovascular disease, or poor response to infection (31). Previous studies have reported that renal failure is closely associated with DFU mortality (25). Notably, A prospective study showed that 40% of patients died during the follow-up period, 35% of those due to renal failure (33). In our study, a total of 7 cases died from CKD with hemodialysis, which similarly suggested that CKD contributed much to mortality of DFUs patients. In addition, 16 patients died from cardiovascular events, indicating that severe systemic comorbidities, such as cardiac or cerebrovascular events as well as renal disease, rather than diabetic foot ulcers itself, may contribute to death of DFUs patients. This most certainly further reduces the attribution of cause away from lower extremity morbidity and towards a more familiar cardio or cerebrovascular and renal etiology (3).

Our results further showed that HCE, Osmolality, HCO3- and HbA1c were the indicators of the humoral environment. It is important to note that, HCE followed closely behind, ranking second, according to the contribution in the model, suggesting that it may be another independent risk factor associated with death of DFUs patients. In addition, Osmolality, HCO3- and HbA1c also contributed as much in this model. Osmolality, HCO3- and HbA1c, as indicators for the severity of diabetic ketoacidosis and hyperglycemic hyperosmolar status, might further exacerbate attribution of HCE in the death for DFUs patients. It has been reported that in patients with severe diabetic hyperosmolarity, the bilateral, symmetrical, and distal extremity involvement suggested diminished blood flow due to hyperviscosity, hypotension, vasoconstrictors, or cholesterol embolism rather than a proximal arterial obstruction as causative mechanisms for lower limb ischemia that resulted in toe dry digital necrosis (26). Meanwhile, emerging evidences indicate that HCE, including DKA, are associated with an inflammatory state marked by elevation of pro-inflammatory cytokines such as tumor necrosis factor-α, and interleukins. Therefore, this procoagulant and inflammatory state may explain the relatively high incidence of thrombotic events observed in HCE. Therefore, we inferred that arterial or venous thrombosis secondary to the hypercoagulable state of HCE and microvascular dysfunction caused by severe dehydration and septic shock, which eventually resulted in severe ischemia of the extremities and diabetic foot occurred or deteriorated. So anticoagulants may be recommended in patients with HCE to protect against diabetic foot ulcer if there are no contraindications of bleeding.

Hemoglobin and albumin can reflect a patient’s nutritional status. Poor nutritional status affects the healing of foot wounds, and exacerbates deterioration of cardiovascular and cerebrovascular diseases, which leading to acute cardiovascular and cerebrovascular events. Thus, promptly examination of the patient coupled with timely correction of the causes are imperative to supporting growth of granulation issue.

The present study has several limitations. First, although we employed internal cross-validation in building the model, it still lacked external validation cohorts and clinical evidence. Secondly, the relative importance ranking of risk factors in the model is not equal to the importance in the causal chain. Thirdly, due to the low incidence of DFUs combined with HCE, the sample size of this study is slightly small, further prospective longitudinal study with larger sample size should be conducted for validation. Fourthly, several confounding factors might have affected our results owing to the fact that the study was conducted at a city designated emergency center, the patients included were almost in a critical condition. Fifthly, due to the low incidence of DFUs combined with HCE, the sample size of DFUs-HCE group was small in this study, we therefore did not evaluate the mortality of patients in different periods. Sixthly, as this is a long-term follow-up study based on retrospective data collection, not all patients could be reviewed regularly, laboratory data during the follow-up period were not included. Notably, more than 50% patients suffered from severe cardiovascular events, such as acute myocardial infarction, congestive heart failure and so on. Furthermore, the follow-up time is not long enough, while the longest follow-up time was 54 months, thus, a longer follow-up should be needed to validate the observed outcomes.

Despite these limitations, to the best of our knowledge, the present study was the firstly to report the clinical outcomes for DFUs concomitant with HCE using traditional statistics and machine learning. Patients with DFUs-HCE had significantly lower immediate survival rates (first 1-6 month) than DFUs alone subjects. In addition, we developed a machine learning model with sufficient accuracy to evaluate and verify relative importance of risk factors of all-cause mortality of HCE overlapping in DFUs patients. We conclude that HCE is an important risk factor for death in DFUs patients. These results need to be further verified in large-scale prospective randomized controlled trials.

## Data availability statement

The original contributions presented in the study are included in the article/supplementary material. Further inquiries can be directed to the corresponding authors.

## Ethics statement

The studies involving human participants were reviewed and approved by the Ethical Committee Board of Chongqing university central hospital. The patients/participants provided their written informed consent to participate in this study.

## Author contributions

LD and PX performed research, analyzed data, and contributed to the discussion and writing of the manuscript. YC, SR, and CY performed research, analyzed data, and contributed to the discussion. BD and MW contributed to the discussion and edition of the manuscript. DA, YM, and WD designed research, analyzed data, contributed to the discussion, and edited the manuscript. WD is the guarantor of this work and, as such, had full access to all the data in the study and takes responsibility for the integrity of the data and the accuracy of the data analysis. All authors contributed to the article and approved the submitted version.

## Funding

This research was funded by the Joint Medical Research Programs of Chongqing Science and Technology Bureau and Health Commission Foundation (No. 2022QNXM018), the Fundamental Research Funds for the Central Universities (2021CDJYGRH-012), the Science and Technology Research Program of Chongqing Municipal Education Commission (Grant No. KJQN201900101). This study is also partially supported by National Institutes of Health, National Institute of Diabetes and Digestive and Kidney Diseases Award Number 1R01124789-01A1 and National Science Foundation (NSF) Center to Stream Healthcare in Place (#C2SHiP) CNS Award Number 2052578 awarded to DA.

## Acknowledgments

The authors thank all of the patients and control subjects for participation in the study.

## Conflict of interest

The authors declare that the research was conducted in the absence of any commercial or financial relationships that could be construed as a potential conflict of interest.

## Publisher’s note

All claims expressed in this article are solely those of the authors and do not necessarily represent those of their affiliated organizations, or those of the publisher, the editors and the reviewers. Any product that may be evaluated in this article, or claim that may be made by its manufacturer, is not guaranteed or endorsed by the publisher.
